# Tropomyosin 2.1 collaborates with fibronectin to promote TGF-β_1_-induced contraction of human lung fibroblasts

**DOI:** 10.1186/s12931-021-01730-y

**Published:** 2021-04-28

**Authors:** Peta Bradbury, Cassandra P. Nader, Aylin Cidem, Sandra Rutting, Dianne Sylvester, Patrick He, Maria C. Rezcallah, Geraldine M. O’Neill, Alaina J. Ammit

**Affiliations:** 1 Woolcock Emphysema Centre, Woolcock Institute of Medical Research, University of Sydney, Sydney, NSW Australia; 2 School of Life Sciences, Faculty of Science, University of Technology Sydney, Sydney, NSW Australia; 3 Respiratory Cellular and Molecular Biology, Woolcock Institute of Medical Research, University of Sydney, Sydney, NSW Australia; 4 Priority Research Centre for Healthy Lungs, Hunter Medical Research Institute and University of Newcastle, Newcastle, Australia; 5 Children’s Cancer Research Unit, Kids Research Institute, Children’s Hospital at Westmead, Sydney, NSW Australia; 6 Children’s Hospital at Westmead Clinical School, Sydney, Australia; 7 School of Medical Sciences, Faculty of Medicine and Health, University of Sydney, Sydney, NSW Australia

**Keywords:** Lung fibrosis, Tropomyosins, Fibronectin, Collagen contraction, Fibroblasts

## Abstract

Many lung diseases are characterized by fibrosis, leading to impaired tissue patency and reduced lung function. Development of fibrotic tissue depends on two-way interaction between the cells and the extra-cellular matrix (ECM). Concentration-dependent increased stiffening of the ECM is sensed by the cells, which in turn increases intracellular contraction and pulling on the matrix causing matrix reorganization and further stiffening. It is generally accepted that the inflammatory cytokine growth factor β_1_ (TGF-β_1_) is a major driver of lung fibrosis through the stimulation of ECM production. However, TGF-β_1_ also regulates the expression of members of the tropomyosin (Tm) family of actin associating proteins that mediate ECM reorganization through intracellular-generated forces. Thus, TGF-β_1_ may mediate the bi-directional signaling between cells and the ECM that promotes tissue fibrosis. Using combinations of cytokine stimulation, mRNA, protein profiling and cellular contractility assays with human lung fibroblasts, we show that concomitant induction of key Tm isoforms and ECM by TGF-β_1,_ significantly accelerates fibrotic phenotypes. Knocking down Tpm2.1 reduces fibroblast-mediated collagen gel contraction. Collectively, the data suggest combined ECM secretion and actin cytoskeleton contractility primes the tissue for enhanced fibrosis. Our study suggests that Tms are at the nexus of inflammation and tissue stiffening. Small molecules targeting specific Tm isoforms have recently been designed; thus targeting Tpm2.1 may represent a novel therapeutic target in lung fibrosis.

## Introduction

In the western world, fibrosis is a major etiological factor in an estimated 45% of deaths [1] and interstitial lung diseases typified by fibrosis, including idiopathic pulmonary fibrosis (IPF), are significant contributors to global disease burden [2]. Increased extracellular matrix (ECM) secretion and deposition by tissue-resident fibroblasts (and other cells) increases tissue stiffness that characterizes fibrosis in IPF [3]. Moreover, fibroblasts pull on the ECM via forces exerted through the cellular actin cytoskeleton and this too can lead to increased tissue stiffening [4]. In previous studies, the inflammatory mediator TGF-β_1_ has been shown to induce ECM deposition [5, 6]. In separate studies, TGF-β_1_ has been shown to increase expression of the actin-associating tropomyosin (Tms) that are required for cells to exert force on the external environment [7]. We sought to determine whether TGF-β_1_ induces concomitant ECM deposition and Tms in human lung fibroblasts, thereby generating a feed-back loop that promotes tissue fibrosis.

Tissue desmoplasia (growth of fibrous or connective tissue) is initiated through large-scale ECM structural deregulation causing increased ligand density, fibril attachment and crosslinking, sustained by unregulated production of growth factors, cytokines and secretion of atypical matrix components. Damaged epithelial, endothelial and smooth muscle cells (and other structural cells) release cascades of inflammatory mediators. These mediators promote increased platelet formation, vasodilation, the stimulation of collagen-secreting fibroblasts and the secretion of matrix-metalloproteinases that degrade and reorganize the ECM. Cells sense the external tissue mechanical features by exerting intracellular-derived forces on the matrix generated by myosin-motor mediated contraction of bundles F-actin (stress fibres) [8]. The stress fibers are coupled via adaptor proteins to the cytoplasmic tail of ECM-bound integrins and ‘pull’ on ECM fibers on the external cell surface through the integrins [9]. The magnitude of the force required to deform the ECM by the cell is therefore related to the ECM stiffness and, in this manner, cells probe and respond to the mechanical forces in local tissue. A key aspect of the fibrotic circuit is the ability of the cells to sense forces in the external environment and to then contribute to the stiffening of the external tissue by relaying corresponding force onto the ECM [4]. Tms decorate the actin filaments and the specific isoform regulates the contractile function of the associated actin filament [10]. The Tm isoform Tpm2.1 is an example of a Tm that stabilizes actin stress fibres and thereby increases the contractile force derived through the stress fibres and exerted onto the surrounding tissue. Previous studies have shown that TGF-β_1_ induces Tpm2.1 [11], but this has not previously been reported in human lung fibroblasts. In this study, we focus on Tpm2.1 (an isoform coded from the TM1 gene) along with other key Tm isoforms associated with actin stabilization coded from the TM1 (Tpm1.6, Tpm1.7) and TM3 (Tpm3.1) gene.

IPF is characterized by excessive ECM deposition causing the lung parenchyma to stiffen, resulting in the progressive loss of lung function. While anti-fibrotic therapeutics, such as nintedanib and pirfenidone, have shown promise [12], a greater understanding of the underlying molecular mechanisms that underpin fibrotic progression may yield novel therapeutic targets. Despite a recognition that fibroblasts play a key role in pathogenesis and tissue dysfunction in pulmonary fibrosis, most studies to date have focused on the molecules and pathways that regulate ECM production, and in particular those induced by the pro-fibrotic inflammatory mediator, transforming growth factor β_1_ (TGF-β_1_). TGF-β_1_ is clearly a therapeutic target in IPF and other fibrotic conditions. However, treatment approaches targeting TGF-β_1_ signaling has proved challenging due to its integral role in health. These obstacles may prove to be overcome by innovative dosing regimens or other interventions [13].

Notably, a recent study revealed that in response to TGF-β_1_, lung fibroblasts showed greater cytoskeletal reorganization and importantly lung fibroblasts from IPF patients were stiffer when compared to control [14], suggesting that increased cellular stiffness may contribute to IPF pathology. Similar studies by Sarna et al. demonstrated change in stiffness of cells derived from people with asthma [15], underscoring the importance of fibrosis in respiratory diseases more broadly. Thus, in this study we test whether, in addition to its well-established ability to induce expression of the ECM fibronectin [5, 6], TGF-β_1_, also increases tropomyosin expression in human lung fibroblasts. We profile tropomyosin isoform and ECM production following TGF-β_1_ treatment and measure the functional outcomes by collagen gel contraction.

## Materials and methods

### Cell culture

Human lung fibroblasts were isolated from the parenchyma of lungs obtained from patients undergoing lung transplantation or surgical resection for thoracic malignancies, in accordance with procedures approved by the Sydney South West Area Health Service. Tables 1 and 2 show patient characteristics. Human lung fibroblasts were dissected and purified as previously described [16, 17]. Primary human fibroblasts were cultured in Dulbecco’s Modified Eagles Medium (DMEM) supplemented with 5% heat-inactivated fetal bovine serum, 0.5 mM L-glutamine, 20 mM HEPES and 1000 units/mL of penicillin, 1000 µg/mL of streptomycin, and 2.5 µg/mL of amphotericin B. All cells were seeded at 1.3 × 10^4^ cells/cm^2^, cultured for 48 h and underwent quiescence (DMEM supplemented with 0.1% bovine serum albumin, 0.5 mM L-glutamine, 20 mM HEPES and 1,000 units/mL of penicillin, 1,000 µg/mL of streptomycin, and 2.5 µg/mL of amphotericin B) for 24 h prior to experimentation. All cell cultures tested negative for mycoplasma prior to experimentation and only cell cultures at less than 6 passages were used.

**Table 1:** Summary of patient demographics (for Fig. 5)

Donor	Diagnosis	Age	Gender	Surgery
1	IPF	63	F	Explanted lung
2	IPF	62	M	Explanted lung
3	Sarcoidosis and pulmonary hypertension	57	M	Explanted lung
4	IPF	55	M	Explanted lung
5	IPF	65	M	Explanted lung
6	IPF	59	M	Explanted lung
7	Rejected lung transplant	61	M	Explanted lung
8	IPF	68	M	Explanted lung
9	IPF	57	M	Explanted lung
10	IPF	64	M	Explanted lung
11	COPD	62	M	Explanted lung
12	COPD	62	M	Explanted lung
13	IPF	65	M	Explanted lung
14	Severe asthma	51	M	Biopsy
15	IPF	59	M	Explanted lung
16	IPF	60	M	Explanted lung
17	COPD	59	M	Explanted lung
18	Pulmonary hypertension	26	F	Biopsy
19	IPF	57	M	Explanted lung
20	COPD	51	M	Explanted lung

COPD, chronic obstructive pulmonary disease; IPF, idiopathic pulmonary fibrosis

**Table 2:** Summary of patient demographics (Fig. 8)

Donor	Diagnosis	Age	Gender	Surgery
1	Interstitial Lung Disease	70	F	Transplant
2	Emphysema	55	M	Transplant
3	Squamous Cell Carcinoma	62	F	Resection
4	Non-Small Cell Carcinoma	60	M	Resection
5	Non-Small Cell Carcinoma	72	M	Resection
6	Lung Mass	62	M	Resection

### Chemicals and reagents

Human recombinant TGF-β_1_ was from BioLegend (San Diego, CA). Type I collagen (rat tail) was from ThermoFisher Scientific (Waltham, MA). Custom-designed siRNA against Tpm2.1 siRNAs were purchased from Qiagen (Germantown, MD), comprising sequences targeting human Tpm2.1 (5′-AAGCACATCGCTGAGGATTCA-3′). Scrambled control sequences for knockdown experiments were Qiagen Allstar Non-targeting Control siRNA (Qiagen). Knockdown was achieved through siRNA transfection with Lipofectamine 2000 (Life Technologies, Carlsbad, CA). Unless otherwise specified, all chemicals used in this study were purchased from Sigma Aldrich (St. Louis, MO).

### Real-time RT-PCR

Total RNA was extracted using the RNeasy Mini Kit (Qiagen) and reverse transcription performed by using the RevertAid First strand cDNA Synthesis kit (Fermentas Life Sciences, Hanover, MD) according to the manufacturer’s protocol. Real-time RT-PCR was performed on an ABI Prism 7500 with fibronectin 1 (Hs01549976_m1) TaqMan gene expression assays and GAPDH (Hs02786624_g1) as the endogenous control probe (Applied Biosystems, Foster City, CA) subjected to the following cycle parameters: 50 °C for 2 min, 1 cycle; 95 °C for 10 min, 1 cycle; 95 °C for 15 s, 60 °C for 1 min, 40 cycles and mRNA expression quantified by delta delta Ct calculations.

### Protein extraction, SDS-PAGE and Western blotting

Adherent cells were lysed in 0.1% sodium dodecyl sulphate-RIPA buffer (containing protease inhibitors), sheared through a 26G syringe and needle, before centrifuging the lysate to remove the pellet. Protein concentration was measured by BCA assay and equal concentrations of protein added to the SDS-PAGE gels (fibronectin (6% gels)—10 µg protein applied; Tm isoforms (6% gels)—5 µg protein applied). Western blotting for fibronectin was performed with mouse monoclonal antibodies (clone IST-4: Sigma-Aldrich), compared to HSP-70 as the loading control (clone BRM-22: Sigma-Aldrich). Tm isoforms were detected with mouse monoclonal antibodies (generously provided by Peter Gunning, University of New South Wales) against Tpm1.6, Tpm1.7 and Tpm2.1 (α/9d) and Tpm3.1 (γ/9d) antibodies [7], compared to α-tubulin as the loading control (clone DM 1A, Santa Cruz Biotechnology). The choice of loading control (i.e. HSP-70 (~ 70 kDa) for fibronectin (~ 200 kDa) and α-tubulin (~ 55 kDa) for TM isoforms (~ 30 kDa)) was dictated by the molecular weight of the protein of interest and the relative mobility in either 6% or 12% SDS-PAGE, respectively, as the Western blots were reprobed for loading controls. Primary antibodies were detected with goat anti-mouse horseradish peroxidase–conjugated secondary antibodies (Cell Signaling Technology, Danvers, MA) and visualized by enhanced chemiluminescence (PerkinElmer, Wellesley, MA). Densitometry was performed with ImageJ [18].

### Collagen gel contraction assay

Collagen gel contraction assays were performed by adapting previous publications [19, 20]. Briefly, 1 × 10^5^ human lung fibroblasts in media (as above) were seeded within 1.3 mg/mL collagen (rat tail Type I) gels. Cells were incubated for 48 h at 37 °C. The cells then underwent quiescence for 24 h at 37 °C and the gels released from the wells by running a sterile yellow tip around the side of the well. Gels were left untreated (control) or stimulated with TGF-β_1_ (2 ng/mL) and images taken at 0, 24, 48, 72 and 96 h using a Kodak Imager. Triplicate technical replicates were performed and collagen gel area at each time point measured by ImageJ [18].

### Tpm2.1 siRNA

Human lung fibroblasts were plated for 24 h before transfection (using Lipofectamine 2000) with siRNA against Tpm2.1 or scrambled control (Non-targeting Control siRNA) (both at a final concentration of 50 nM) for 24 h, as per previous publication [21]. Cells then underwent collagen gel contraction assay (as above) to determine the impact of knocking down Tpm2.1 on contraction at 0, 24, 48, 72 and 96 h. Knockdown was confirmed by Tpm2.1 Western blot (compared to HSP-70 as a loading control) and densitometry performed independently for all experiments.

### Statistical analysis

Statistical analysis was performed with GraphPad Prism 8 using Student's unpaired *t* test and two-way ANOVA then Bonferroni’s multiple comparison test or multiple linear regression (least squares). *P* values < 0.05 were sufficient to reject the null hypothesis for all analyses. A 3D scatter plot was generated in R.

## Results

### TGF-β_1_ increases fibronectin mRNA expression and protein expression

**Fig. 1:**
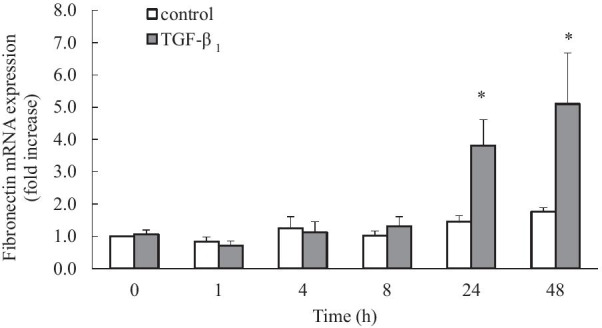
TGF-β_1_ increases fibronectin mRNA expression. Human lung fibroblasts were untreated (control) or stimulated with TGF-β_1_ (2 ng/mL) for 0, 1, 4, 8, 24 and 48 h. Fibronectin mRNA expression was quantified by real-time RT-PCR (results expressed as fold increase compared to control at 0 h). Statistical analysis was performed using two-way ANOVA then Bonferroni's multiple comparisons test (where * denotes a significant effect of TGF-β_1_ on fibronectin mRNA expression (*P* < 0.05)). Data are mean ± SEM values from n = 4–11 human lung fibroblast primary cell cultures

**Fig. 2:**
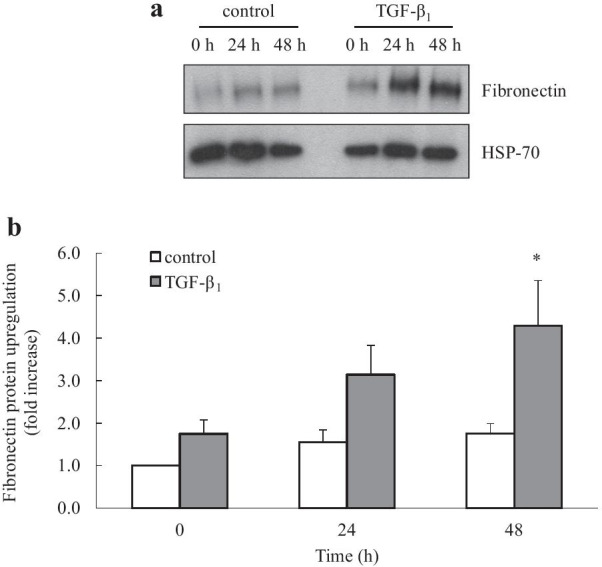
TGF-β_1_ increases fibronectin protein upregulation. Human lung fibroblasts were left untreated (control) or stimulated with TGF-β_1_ (2 ng/mL) for 0, 24 and 48 h. Fibronectin protein upregulation was detected by Western blotting (compared to HSP-70 as a loading control). Results are shown as: **a** a representative Western blot; **b** densitometric analysis of fibronectin protein upregulation (normalised with HSP-70 and expressed as fold increase compared to control at 0 h). Statistical analysis was performed using two-way ANOVA then Bonferroni's multiple comparisons test (where * denotes a significant effect of TGF-β_1_ on fibronectin protein upregulation (*P* < 0.05)). Data are mean ± SEM values from n = 7 human lung fibroblast primary cell cultures

While it is well established that the cytokine TGF-β_1_ increases fibronectin expression in human lung fibroblasts [5, 6], our first aim was to determine the temporal kinetics of TGF-β_1_-induced fibronectin mRNA expression in human lung fibroblasts. These are important foundational experiments to establish the conditions for further exploration. Cells were either untreated (vehicle control) or stimulated with TGF-β_1_ (2 ng/mL) for 0, 1, 4, 8, 24, and 48 h and fibronectin mRNA expression was quantified by real-time RT-PCR. No significant increase in fibronectin mRNA was detected within the first 8 h of TGF-β_1_ stimulation (Fig. 1). However, following TGF-β_1_ stimulation for 24 h a significant increase in fibronectin mRNA expression was observed (3.8 ± 0.8-fold) and sustained at 48 h (5.1 ± 1.6-fold) (*P* < 0.05) (Fig. 1). To determine if TGF-β_1_ also induced fibronectin protein expression, Western blot analysis was conducted following 24 and 48 h of TGF-β_1_ stimulation. Untreated cells show a small, but not significant, increase in fibronectin protein over time in untreated cells (Fig. 2). In comparison, TGF-β_1_ induced an increase in fibronectin protein at both 24 and 48 h post-stimulation (Fig. 2a) and densitometric analysis confirmed that TGF-β_1_ significantly increased fibronectin protein upregulation at 48 h (4.3 ± 1.1-fold) (*P* < 0.05) (Fig. 2b).

### TGF-β_1_ induces Tpm1.6/1.7 and Tpm2.1 protein expression

**Fig. 3:**
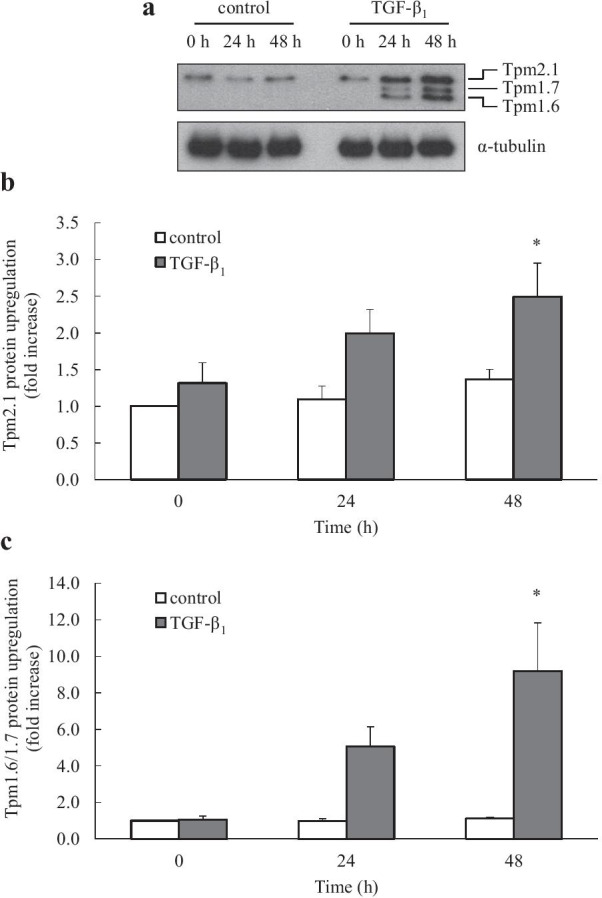
TGF-β_1_-induces protein upregulation of Tpm1.6/1.7 and Tpm2.1, but not Tpm3.1. Human lung fibroblasts were left untreated (control) or stimulated with TGF-β_1_ (2 ng/mL) for 0, 24 and 48 h. Tpm1.6/1.7 and Tpm2.1 protein upregulation was detected by Western blotting with the α/9d antibody, while Tpm3.1 was detected with the γ/9d antibody (compared to α-tubulin as a loading control). Results are shown as: **a** a representative Western blot; densitometric analysis of (**b**) Tpm2.1 and (**c**) Tpm1.6/1.7 protein upregulation (normalised with α-tubulin and expressed as fold increase compared to control at 0 h). Statistical analysis was performed using two-way ANOVA then Bonferroni's multiple comparisons test (where * denotes a significant effect of TGF-β_1_ on tropomyosin isoform upregulation (*P* < 0.05)). Data are mean ± SEM values from n = 7 human lung fibroblast primary cell cultures

**Fig. 4:**
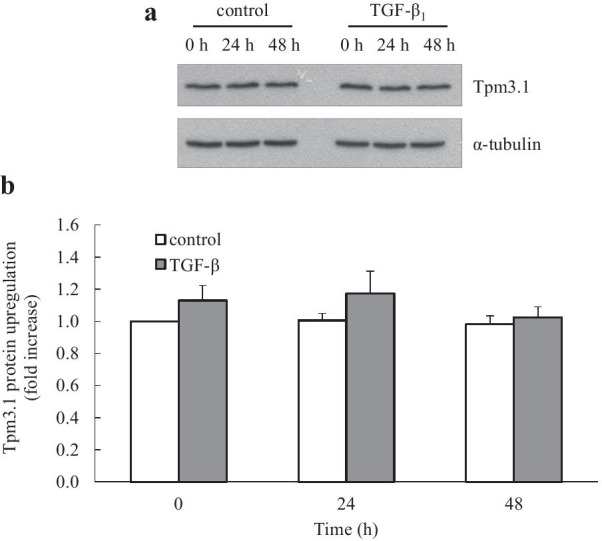
TGF-β_1_ does not induce upregulation of Tpm3.1 protein. Human lung fibroblasts were left untreated (control) or stimulated with TGF-β_1_ (2 ng/mL) for 0, 24 and 48 h. Tpm3.1 protein upregulation was detected by Western blotting with the γ/9d antibody (compared to α-tubulin as a loading control). Results are shown as: **a** a representative Western blot; **b** densitometric analysis of Tpm3.1 protein upregulation (normalised with α-tubulin and expressed as fold increase compared to control at 0 h). Data are mean ± SEM values from n = 10 human lung fibroblast primary cell cultures

The impact of TGF-β_1_ on the multi-gene family of tropomyosins in this cell type was unknown. We address this herein, by performing Western blotting with two monoclonal antibodies: α/9d and γ/9d that detect specific isoforms coded by the TM1 (Tpm1.6, Tpm1.7 and Tpm2.1) and TM3 (Tpm3.1) genes, respectively. Human lung fibroblasts were treated with TGF-β_1_ or vehicle (control) for 24 and 48 h. Control primary fibroblasts showed no change in Tpm2.1 expression, and also showed no Tpm1.6 or Tpm1.7 expression over the time course (Fig. 3a). Conversely, TGF-β_1_-stimulated fibroblasts showed increased expression of Tpm2.1, Tpm1.6 and Tpm1.7 at both 24 and 48 h (Fig. 3a). Densitometric analysis revealed that stimulation with TGF-β_1_ for 48 h induced a significant 2.5-fold increase in Tpm2.1 (Fig. 3b) and 9.2-fold increase in Tpm1.6/1.7 isoforms (Fig. 3c; Tpm1.6 and Tpm1.7 isoforms were too close to analyze separately by densitometry and have therefore been grouped together and referred to as Tpm1.6/1.7) (*P* < 0.05). In contrast, Western blotting with γ/9d showed that TGF-β_1_ did not induce upregulation of Tpm3.1 (Fig. 4). Taken together, we show for the first time in human lung fibroblasts that TGF-β_1_ induces the protein expression of both fibronectin and those Tm isoforms encoded by the TM1 gene.

### TGF-β_1_ induces human lung fibroblast-mediated collagen gel contraction

**Fig. 5:**
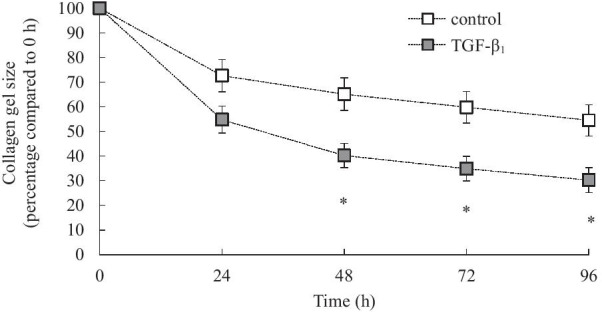
TGF-β_1_ induces human lung fibroblast-mediated collagen gel contraction. Collagen gel contraction assays mediated by human lung fibroblasts were performed by stimulating collagen gels with TGF-β_1_ (2 ng/mL) for 0, 24, 48, 72 and 96 h, in comparison to untreated controls. Results are expressed as collagen gel size (percentage compared to 0 h). Statistical analysis was performed using two-way ANOVA then Bonferroni's multiple comparisons test (where * denotes a significant effect of TGF-β_1_ on human lung fibroblast-mediated collagen gel contraction (*P* < 0.05)). Data are mean ± SEM values from n = 9 human lung fibroblast primary cell cultures

The ability of human lung fibroblasts to contract three-dimensional collagen gels is an in vitro model of ECM remodelling, a functional characteristic of lung fibrosis in vivo [22, 23]. To investigate the effects of TGF-β_1_ on human lung fibroblast-mediated collagen gel contraction, primary fibroblasts from n = 9 donors with a range of diagnoses (see Table 1) were used. Over the 96 h time course, fibroblasts stimulated with TGF-β_1_ displayed significantly increased contraction of the collagen gels from 48 h when compared to the vehicle control (Fig. 5: *P* < 0.05).

### TGF-β_1_-induced upregulation of fibronectin, Tpm1.6/1.7 and Tpm2.1 protein: inter-donor variability

**Fig. 6:**
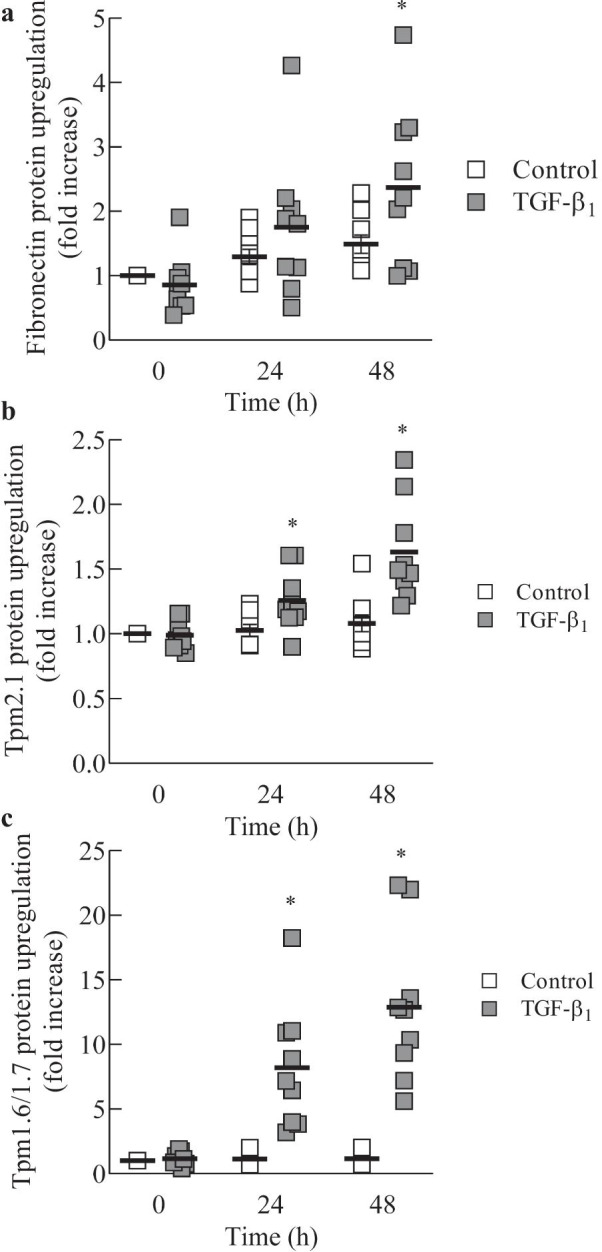
TGF-β_1_-induced upregulation of fibronectin, Tpm1.6/1.7 and Tpm2.1 protein: inter-donor variability. Human lung fibroblasts were left untreated (control) or stimulated with TGF-β_1_ (2 ng/mL) for 0, 24 and 48 h. Western blotting was utilized to quantitate protein levels of: **a** fibronectin (compared to HSP-70 as a loading control); **b** Tpm2.1 (compared to α-tubulin as a loading control); **c** Tpm1.6/1.7 (compared to α-tubulin as a loading control)). Results shown are densitometric analysis (normalised to loading controls and expressed as fold increase compared to control at 0 h). Data are protein levels detected in human lung fibroblasts from each of the nine donors with mean represented as black bars. Statistical analysis was performed using two-way ANOVA then Bonferroni's multiple comparisons test (where * denotes a significant effect of TGF-β_1_ (*P* < 0.05))

While TGF-β_1_-induced fibronectin is a known contributor to the fibrotic lung fibroblast phenotype [23], the role TGF-β_1_-induced Tm expression plays in regulating collagen gel contraction was next assessed. Thus, parallel Western blot experiments were conducted using the same primary human lung fibroblasts used for the collagen gel contraction assay (Fig. 5). Fibroblasts were either left untreated (vehicle control) or stimulated with TGF-β_1_ for 0, 24 and 48 h. Protein levels of fibronectin (Fig. 6a), Tpm2.1 (Fig. 6b), Tpm1.6/1.7 (Fig. 6c) were quantitated and analysed by densitometry. The results are expressed as protein fold change compared to control t = 0 and are expressed for each donor (as well as the mean for the n = 9 donors at each time point). Although there was a significant impact of TGF-β_1_ on resultant protein levels (*P* < 0.05), not surprisingly, there was inter-donor variability between the TGF-β_1_-induced responses with TGF-β_1_ stimulating the fibroblast production of both fibronectin and Tms to different extents (Fig. 6). The biological variability is unsurprising given the range of disease diagnoses in the group of cell donors (Table 1). It is also important to note that the goal of the study was not to link disease to the resultant responses (and the study is not powered to do so). Rather, our aim was to examine cooperation between Tms and fibronectin on contractility of human lung fibroblasts. Accordingly, we next examined the possibility that TGF-β_1_-induced collagen gel contraction may be greater if TGF-β_1_ stimulation upregulates protein production of both fibronectin and TM1 isoforms.

### Tpm2.1 collaborates with fibronectin to promote TGF-β_1_-induced contraction of human lung fibroblasts

**Fig. 7:**
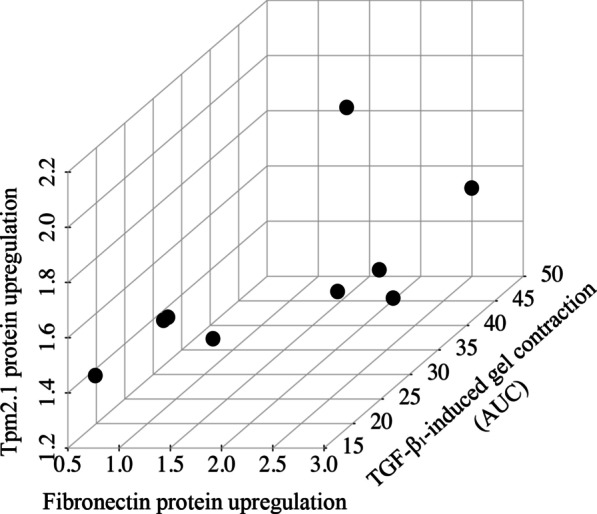
Tpm2.1 collaborates with fibronectin to promote TGF-β_1_-induced contraction of human lung fibroblasts. The relationship is represented graphically in a 3D scatter plot with the z-axis—TGF-β_1_-induced collagen gel contraction (area under the curve: AUC); x-axis—TGF-β_1_-induced fibronectin protein upregulation at 48 h; y-axis—TGF-β_1_-induced Tpm2.1 protein upregulation at 48 h

To address the possibility that fibrosis severity may be linked to the donor’s fibroblast response to TGF-β_1_ stimulation, we performed multiple linear regression to test the hypothesis that fibronectin may collaborate with one or more TM1 isoforms to regulate TGF-β_1_-induced collagen gel contraction. To do this, we represented the collagen gel contraction (from Fig. 5) as TGF-β_1_-induced collagen gel contraction (fold change over control at same time point) and measured area under the curve (AUC). With TGF-β_1_-induced collagen gel contraction (AUC) as the dependent variable, we conducted multiple linear regression to examine whether there was a statistically significant relationship between the dependent variable (contraction) and the explanatory variables (i.e. fibronectin and Tm isoforms) induced by TGF-β_1._ The model is represented as: Y = β_0_ + β_1_*B + β_2_*C; where Y is TGF-β_1_-induced collagen gel contraction (AUC); β_0_ is the intercept (constant term)_;_ β_1_ is the coefficient relating to B—TGF-β_1_-induced fibronectin protein upregulation at 48 h; β_2_ is the coefficient relating to C—TGF-β_1_-induced Tpm1.6/1.7 or Tpm2.1 protein upregulation at 48 h). There was no significant relationship (*P* = 0.5578) between TGF-β_1_-induced fibronectin and Tpm1.6/1.7 upregulation and resulting contraction (Y = 32.52 + 2.26*B – 0.38*C). In contrast, the combination of fibronectin and Tpm2.1 significantly predicted the extent of TGF-β_1_-induced contraction (Y = 56.99 + 9.174*B – 27.02*C: *P* = 0.0221). Thus, fibronectin together with Tpm2.1, but not Tpm1.6/1.7, promotes TGF-β_1_-induced contraction of human lung fibroblasts. This cooperative relationship is represented graphically in a 3D scatter plot (Fig. 7).

### Knocking down Tpm2.1 reduces human lung fibroblast-mediated collagen gel contraction

**Fig. 8:**
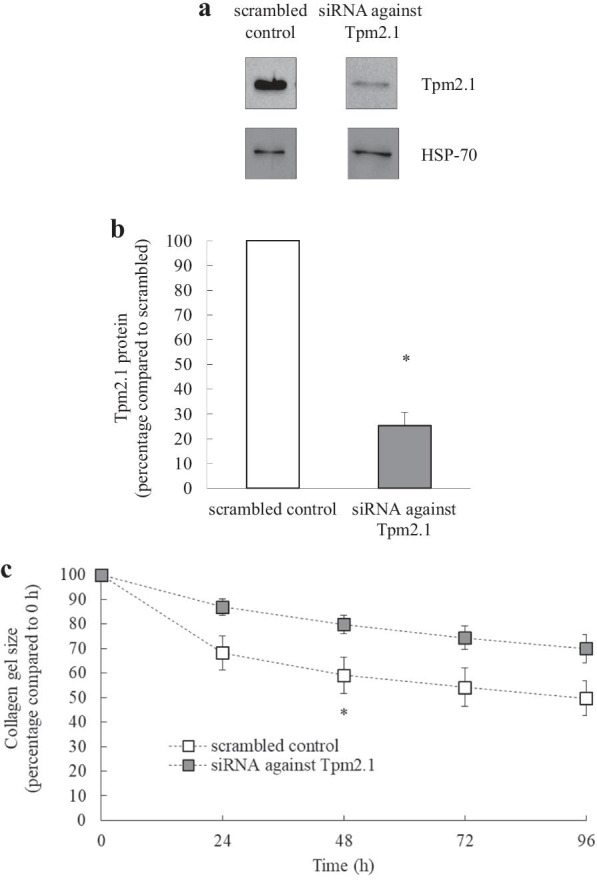
Knocking down Tpm2.1 reduces human lung fibroblast-mediated collagen gel contraction. Human lung fibroblasts were transfected with siRNA against Tpm2.1 or scrambled control and collagen gel contraction measured at 0, 24, 48, 72 and 96 h. Tpm2.1 knockdown at 96 h was confirmed by Western blotting (compared to HSP-70 as a loading control). Results are shown as: **a** a representative Western blot; **b** densitometric analysis of Tpm2.1 protein (normalised with HSP-70 and expressed as percentage of scrambled control); (**c**) collagen gel size (percentage compared to 0 h). Statistical analysis was performed using (**b**) Student's unpaired *t* test and (**c**) two-way ANOVA then Bonferroni's multiple comparisons test (where * denotes that significantly knocking down Tpm2.1 protein represses human lung fibroblast-mediated collagen gel contraction (*P* < 0.05)). Data are mean ± SEM values from n = 6 human lung fibroblast primary cell cultures

To demonstrate the importance of Tpm2.1 in human lung fibroblast-mediated collagen gel contraction, primary fibroblasts from n = 6 donors with a range of diagnoses (see Table 2) were transfected with siRNA against Tpm2.1 or scrambled control for 24 h. Cells then underwent collagen gel contraction assay and gel size in cells transfected with scrambled control or siRNA against Tpm2.1 measured at 0, 24, 48, 72 and 96 h. Knockdown was confirmed by Western blotting and, as shown in Figs. 8a and b, siRNA against Tpm2.1 reduced Tpm2.1 protein by 74.7 ± 5.3%, compared to scrambled control. Over the 96 h time course, knocking down Tpm2.1 significantly reduced human lung fibroblast-mediated collagen gel contraction, when compared to the scrambled control (Fig. 8c: *P* < 0.05).

## Discussion

Fibrotic tissue remodeling has a significant impact on morbidity and mortality worldwide. Treatment options are limited, and the fundamental mechanisms of disease progression are poorly understood. Thus, there is an urgent need to investigate fibrosis at the cellular and molecular level. The expression pattern of tropomyosin isoforms is critical, as the pattern of tropomyosin isoform decoration of actin filaments is a key determinant of the dynamics of the associated actin filament. We have shown that TGF-β_1_ induces the expression of Tpm1.6/1.7 and 2.1 and that via concomitant induction of Tpm2.1 and fibronectin protein expression, TGF-β_1_ induces human lung fibroblast-mediated collagen gel contraction.

During the fibrotic process, the actin cytoskeletons of fibroblasts in the tissue play a twofold role. Firstly, inflammatory cytokines induce enlarged actin fibers and focal adhesions. Secondly, the cells sense the increasing rigidity of the ECM by exerting traction forces through their focal adhesions (mechanosensing) and then convert this rigidity signal into increased actin fiber formation through the activation of biochemical signaling pathway (mechanotransduction) [24]. Increased actin fiber formation enables the cells to exert greater forces through the focal adhesions, which contracts the ECM and thereby disrupts the tissue structure [25]. Collectively, these changes to the fibroblast cellular structure therefore underpin the development of fibrosis. This interface of inflammation and mechanobiology is a key determinant of cellular architecture and tissue functioning. Currently, we do not fully understand why cells tip over into aberrant actin filament and focal adhesion formation to create the stiff, immobile fibroblast cells that are characteristic of fibrotic tissue. Intriguingly, a recent study by Jaffar et al*.* [14] demonstrated greater cellular stiffness in fibroblasts from people with IPF and greater cytoskeletal reorganization in response to TGF-β_1_. We propose that tropomyosins are key to actin stabilisation that characterize fibroblasts in fibrotic tissue.

Tropomyosins are a multi-isoform family of proteins that assemble into polymers and lay in the major groove of polymerized actin filaments [10]. The association of the tropomyosin polymer with actin filaments in turn determines the association of molecules that control actin filament turnover [26–28]. Ultimately, the pattern of tropomyosin decoration of actin filaments defines the function of the associated filament [29, 30]. The tropomyosin isoforms we have examined in this study have been associated with actin stabilisation, a key characteristic of cellular stiffening that occurs in fibrosis. Tpm1.6 stabilises stress fibres [31] and Tpm1.6/1.7 was recently shown to be recruited to actin filaments to increase actomyosin contractility [32]. Notably, the actin stabilizing role of isoform Tpm2.1 (previously known as Tm1) has previously led some investigators to describe it as a pro-fibrotic response gene [33]. Tpm3.1 (previously known as Tm5NM1) has been shown to stimulate actin stress fibre formation due to actin stabilisation [26, 34] in the context of cancer cell migration. Tm isoforms can exert distinct effects that depend on the cell’s physical environment (such as ECM) and thus it is important to note that cell- and disease-type differences can exist.

Tropomyosins are key players in actin dynamics and despite the key role of the actin cytoskeleton influencing fibrosis, surprisingly few studies have investigated the role of the pro-fibrotic cytokines TGF-β_1_ on tropomyosin expression, and none in lung fibroblasts from human subjects. Most studies to date have focused on the role and regulation of tropomyosins in TGF-β_1_-induced epithelial-mesenchymal transition in transformed epithelial cell lines (such as A549 [35], NMuMG [33, 36]) or primary lens epithelium [37, 38]. Schevzov et al*.* have shown that expression of Tm isoforms in human and mouse lung fibroblasts [7] and we are the first to extend these studies in human lung fibroblasts to show the inflammatory cytokine TGF-β_1_ also induce Tpm1.6/1.7, Tpm2.1, but not Tpm3.1.

We examined the hypothesis that cooperation exists between tropomyosin isoforms upregulated by TGF-β_1_ and fibronectin on the resultant collagen gel contraction, a model of lung stiffening [23]. We observed variability in patient specific expression of fibronectin, Tpm1.6/1.7 and Tpm2.1 induced by TGF-β_1_ (at 48 h) from n = 9 donors. Interestingly, multiple linear regression demonstrated a statistically significant relationship between the extent of Tpm2.1 and fibronectin induced by TGF-β_1_ and resultant collagen gel contraction. This would align with the known function of Tpm2.1 to stabilise actin and promote fibrosis [33], in addition to known ability of TGF-β_1_ to synthesise and deposit fibronectin into surrounding remodelled environment. Taken together, our results link Tpm2.1 to lung fibroblast stiffening that characterises fibrosis in IPF. However, while Fig. 7 offers a correlation between fibronectin, Tpm2.1 and TGF-β_1_-induced contractility, correlation need not mean causation. We conducted further experimentation (Fig. 8) to show that knocking down Tpm2.1 reduces human lung fibroblast-mediated collagen gel contraction.

## Conclusion

At the interface of inflammation and mechanobiology, we propose that tropomyosins may play a role in controlling fibrotic development. Excitingly, selective Tm inhibitors are now in development [7, 39, 40], but their successful use in lung fibrosis first requires an in depth understanding of the inflammation-mediated regulatory networks that control tropomyosin isoform expression in lung fibroblasts. As modulation of tropomyosin isoform expression represents a tuneable approach towards controlling actin dynamics and cytoskeletal reorganization, the new knowledge gained by our study is essential for developing anti-fibrotic pharmacotherapeutic strategies for interstitial lung diseases in the future. Given that knocking down Tpm2.1 reduces human lung fibroblast-mediated collagen gel contraction, our data support the assertion that tropomyosins are tuneable actin-binding proteins at the interface of inflammation and mechanobiology and suggest that targeting Tpm2.1 may represent a therapeutic target in lung fibrosis.

## Data Availability

Available upon request.
